# Evaluation of limited blood sampling population input approaches for kinetic quantification of [^18^F]fluorothymidine PET data

**DOI:** 10.1186/2191-219X-2-11

**Published:** 2012-03-24

**Authors:** Kaiyumars B Contractor, Laura M Kenny, Charles R Coombes, Federico E Turkheimer, Eric O Aboagye, Lula Rosso

**Affiliations:** 1 Department of Surgery and Cancer, Imperial College Healthcare NHS Trust, Hammersmith Hospital, London, UK; 2 Department of Neuroscience, Imperial College London, London, UK; 3 Imperial College School of Medicine, Room 240, MRC Cyclotron Building, Clinical Sciences Centre, Hammersmith Hospital, Du Cane Road, London W12 0NN, UK

**Keywords:** FLT, Kinetic modelling, Input function, Quantification, Tumour proliferation

## Abstract

**Background:**

Quantification of kinetic parameters of positron emission tomography (PET) imaging agents normally requires collecting arterial blood samples which is inconvenient for patients and difficult to implement in routine clinical practice. The aim of this study was to investigate whether a population-based input function (POP-IF) reliant on only a few individual discrete samples allows accurate estimates of tumour proliferation using [^18^F]fluorothymidine (FLT).

**Methods:**

Thirty-six historical FLT-PET data with concurrent arterial sampling were available for this study. A population average of baseline scans blood data was constructed using leave-one-out cross-validation for each scan and used in conjunction with individual blood samples. Three limited sampling protocols were investigated including, respectively, only seven (POP-IF7), five (POP-IF5) and three (POP-IF3) discrete samples of the historical dataset. Additionally, using the three-point protocol, we derived a POP-IF3M, the only input function which was not corrected for the fraction of radiolabelled metabolites present in blood. The kinetic parameter for net FLT retention at steady state, K_i_, was derived using the modified Patlak plot and compared with the original full arterial set for validation.

**Results:**

Small percentage differences in the area under the curve between all the POP-IFs and full arterial sampling IF was found over 60 min (4.2%-5.7%), while there were, as expected, larger differences in the peak position and peak height.

A high correlation between K_i _values calculated using the original arterial input function and all the population-derived IFs was observed (R^2 ^= 0.85-0.98). The population-based input showed good intra-subject reproducibility of K_i _values (R^2 ^= 0.81-0.94) and good correlation (R^2 ^= 0.60-0.85) with Ki-67.

**Conclusions:**

Input functions generated using these simplified protocols over scan duration of 60 min estimate net PET-FLT retention with reasonable accuracy.

## Background

Positron emission tomography (PET) imaging with [^18^F]fluorothymidine (FLT-PET) is believed to provide an *in vivo *measurement of tumour proliferation [1]. FLT is a substrate for the cell-cycle dependent enzyme, thymidine kinase (TK1), and its accumulation in cells is proportional to the activity of this enzyme, which in turn is correlated with sustained cellular proliferation. The accumulation of FLT in cells, therefore, provides an indication of their proliferation rate [2], and a high positive correlation with Ki-67 labelling index (Ki-67), an immunohistochemical marker for cellular proliferation, has been found [3-6]. Although semi-quantitative measures, like the standard uptake value (SUV), can be obtained from both static and dynamic PET images, only a full kinetic analysis will provide direct information on the rate of FLT phosphorylation. Importantly, a quantitative measure of tumour FLT flux may be necessary when evaluating novel anti cancer therapies for which the effect on kinetics is unknown.

Key physiological parameters of PET tracer kinetics can be quantified using models which usually require an input function (IF)--a measure of the amount of radioactivity that enters the organ of interest. As FLT undergoes glucuronidation predominantly in the liver, the fraction of the FLT-glucuronide radiolabelled metabolite present in plasma needs also to be monitored and used to derive the metabolite corrected input function. Peripheral arterial blood sampling during the time of the scan has been extensively used for this purpose and has been shown to achieve the desirable precision in modelling the kinetics of radiotracers. In order to obtain an accurate profile of radioactive blood concentration in the first 10 min after tracer injection, continuous sampling is often used. However, arterial cannulation has a number of drawbacks which limit the number of blood samples that could be obtained and prohibit the use of continuous sampling in some groups, particularly in cancer patients who are often anaemic. This fact, combined with the complexity and high cost of PET measurements, makes it highly desirable to validate alternative approaches for obtaining input functions and maximise the information obtained.

When the PET images include regions of activity of a prominent blood vessel, like the aorta, these may be sampled to derive a blood pool IF that can be used as replacement for continuous sampling for the early period when radioactive spill over from surrounding tissue is limited. However, because of the small size of these regions, which is often inferior or comparable to the resolution of the PET scan, the image-derived input function methods require careful correction for partial volume effects [7-11] and still necessitate a number of blood samples for metabolites analysis if the tracer is metabolised during the scan.

Recently, there has been interest in the use of limited arterial blood sampling methods [12-14] that explore the possibility of using fewer samples, albeit with a loss of accuracy, but leading to better clinical compliance. Additionally, to avoid individual continuous sampling and account for blood sampling errors, a population-based input function may be determined. This involves pooling together previously collected arterial blood data and calculating the average blood response to radiotracer injection. Subject specific calibration of the mean blood curve can be performed using a few individual discrete blood samples. The population averaged input function has been described in neurological and some oncology studies using FDG and shows promise [15-18].

To date, relatively few limited sampling methodologies (four to eight samples for blood radioactivity concentration and at least one sample for metabolite analysis) have been proposed for FLT-PET [14,19-22], and FLT kinetics has been recently estimated using image-derived input function in colorectal cancer [21], non-small-cell lung cancer [22] and high grade gliomas [23]. In this study, we investigated the consequences of using a population input approach and highly reduced sampling protocols to estimate FLT kinetics in oncology PET studies without continuous arterial sampling. Previously acquired FLT-PET scans with concurrent continuous arterial data were used to validate the results. We then assessed whether test-retest variability and accuracy for determination of proliferation of the population input functions were affected by this reduced sampling population-based methodology.

## Methods

### FLT data set

The data set comprised of 15 breast cancer patients who underwent three dynamic FLT PET scans. Of the 15 cases, 9 cases underwent two pre-treatment scans within 2-8 days of each other before starting chemotherapy treatment. Baseline tumour proliferation indices Ki-67 were available in 12 of the 15 patients with suitable histology. In the present study, only the primary lesion was analysed for each patient. The reproducibility, as well as correlation with Ki-67, has been previously published [5,24]. The study was approved by the local Hospital Ethics committee and the Administration of Radioactive Substances Advisory Committee, United Kingdom. All patients gave written informed consent to take part.

### Blood data acquisition and analysis of original data set

Patients were scanned in the Siemens ECAT® 962 HR^+ ^PET scanner (CTI/Siemens, Knoxville, TN, USA)). Dynamic PET scanning was commenced after injection of an average of 370 MBq of FLT as a bolus and lasted 90 min. Arterial blood sampling was performed continuously for the first 10 min of scanning, and then discrete arterial samples were taken at baseline and after 2.5, 5, 10, 20, 30, 45, 60, 75 and 90 min. Continuous blood sampling was counted in an in-house bismuth-germanate-oxide counter to record the initial blood sampling radioactivity. For the discrete samples, the total blood radioactivity and plasma radioactivity were determined by gamma counter (Raytek, Sheffield, UK), and the plasma parent (FLT) fraction was determined by HPLC with radiochemical detection (Acrodisc, VWR International Ltd., Leicestershire, UK) [5,24] at 2.5, 5, 10, 30, 45, 60, 75 and 90 min after scan start. All data were fitted using non-linear least square minimisation using Matlab^® ^based on the interior-reflective Newton method [25,26]. The analytical functions for the optimisation were chosen as the one that produced the best agreement, for each given reduced sampling protocol, with the original blood fits. All blood and plasma data belonging to the same type of POP-IFs were fitted using the same functions to ensure consistency within the protocol. The parent fraction was modelled using a sigmoid function, the plasma over blood ratio using an exponential approach to a constant (only the first 10 min), and the whole series of plasma and blood data points using a cubic spline interpolation, see Appendix. Although FLT kinetic constants can be determined with more accuracy using 90 min of dynamic acquisition [27], 60 min acquisition have been used to quantify accurately FLT retention at steady state (K_i_) [14] and would be more advantageous in routine clinical settings. Only 0-60 min arterial blood data of the available 90 min was used for this validation study (i.e. continuous sampling plus seven discrete samples up to 60 min, henceforth, referred to as the "full arterial sampling" set or FA).

### Generation of the population input function

**Figure 1:**
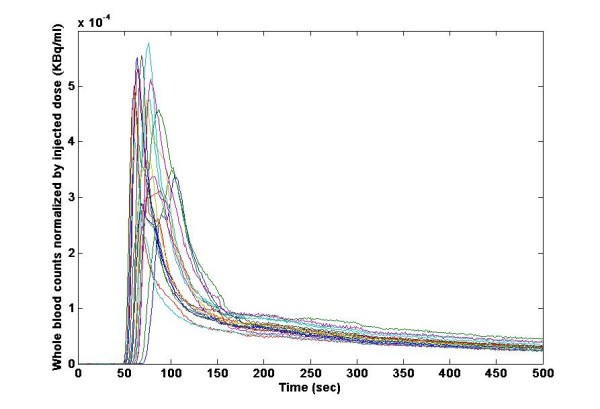
**Whole blood time curves from 36 FLT scans normalised by injected dose**. Arterial blood sampling was performed continuously for the first 10 min of scanning. Note the population variance.

Only the total blood IFs of pre-treatment scans were used to derive the initial part of the POP-IFs (up to 2.5 min) for both pre-treatment and post-treatment scans. Each blood input function was divided by the individual scan injected dose, and to avoid information overlap, we excluded for each patient his/her relative original blood input function(s) and constructed an individualised average normalised total blood input function (POP-IF) for each patient, see Figure 1. Then for each scan, a new population-based parent plasma IF was generated by multiplying the individual scan injected dose of FLT by the dose-normalised average of the leave-one-out pre-treatment subset and corrected by the plasma over blood ratio up to 2.5 min, see Appendix. Correction by subject weight has also been considered, but did not influence the results in this dataset.

The latter part of the POP-IF, after the first sampling data point, was derived by interpolating the individual plasma radioactivity concentration measurements and scaling it by the unchanged FLT fraction modelled using one of three reduced blood sampling protocols as described in the next paragraph. Only in the case of the POP-IF3M the metabolite correction was not performed.

### Reduced blood sampling protocols

To test whether adequate kinetic estimation could be obtained using IF corrected by fewer blood samples, three reduced sets of discrete blood sample data were formulated. The original blood data was abbreviated and several combinations were tested to determine the optimal time points that would best reproduce the original arterial input in a reduced sampling scenario. A full optimization of the sampling times was outside of the scope of this study based on historical data. The first set consisted of all seven discrete samples (up to 60 min) from the original full arterial sampling set, namely at baseline, 2.5, 5, 10, 20, 30, 45 and 60 min post injection of FLT. The population-based input function we derived using this protocol was called POP-IF7. The second set consisted of only five discrete arterial blood samples taken from the original set at 2.5, 5, 10, 30 and 60 min after the start (POP-IF5), and the third set comprised of three samples taken from the original set at times 2.5, 10 and 45 min after start of FLT (POP-IF3). The plasma over blood ratio was modelled using a least square fit to an exponential function leading to a constant to correct the average blood curve. The plasma radioactivity concentration as a function of time were interpolated using a least square fit to a two exponential function plus a constant when three points were available, and a cubic spline plus a constant when five points were available. The resulting plasma input function was corrected for the presence of metabolites. The parent fraction curve was modelled using an exponential approach to a constant when only three points were used, and a sigmoid when five points were available, see Appendix.

Finally, we evaluated the possibility of further simplifying the blood protocol, generating a population-based input function following the same procedure of the POP-IF3 but uncorrected for the contribution of metabolites. This input function was called POP-IF3M. A summary of all population-based input functions is presented in Table 1. Quantification of the differences in the population-derived input functions with respect to the original full arterial sample procedure (i.e. continuous sampling for the first 10 min concurrent with seven discrete samples up to 60 min) was determined by calculating the area under the curve (AUC), height of the peak and position of the peak (with respect to time) for all population-based input functions.

**Table 1:** Summary of blood measurements used to generate the population input functions evaluated in this study

	FA-IF	POP-IF7	POP-IF5	POP-IF3	POP-IF3M
**Continuous sampling**	yes	no	no	no	no
**Number of blood samples**	7	7	5	3	3
**Number of metabolite measures**	7	7	5	3	0

### Data analysis

We wanted to assess how the POP-IFs affected the quantification of tumour FLT-PET uptake compared to using the original FA-IFs derived from the full arterial sampling standard approach. The kinetics of FLT were determined by calculating the net irreversible plasma to tumour transfer constant at steady state, K_i_, the relevant kinetic parameter reflecting cellular proliferation [4]. This was done for all the four population-based input functions using the established modified Patlak's graphical method [5,28] The percentage difference (± standard deviation) between the population-based input functions and full arterial results was calculated, together with the within-subject variability and correlation with Ki-67.

## Results and discussion

### Population input function

**Figure 2:**
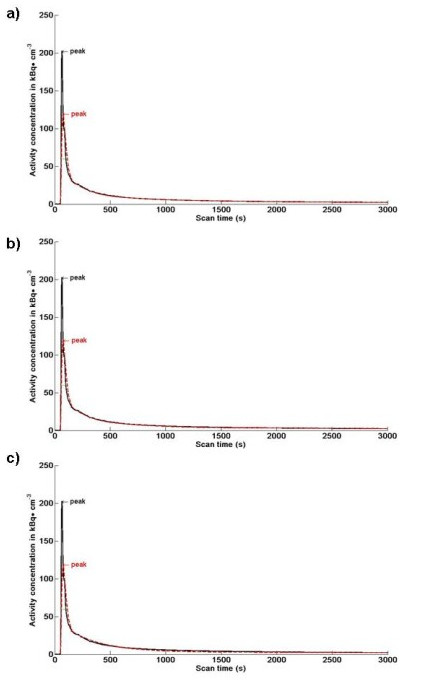
**The comparison of subject 1 input function obtained using full arterial sampling and average POP-IFs**. Comparison of subject 1 input function obtained using full arterial sampling (black curve) and average POP-IF (red curve) obtained using **a**) seven discrete samples up to 65 min (POP-IF7), **b**) five discrete samples (POP-IF5) and **c**) three discrete samples (POP-IF3). As expected, the population-based input functions failed to reproduce the true initial peak height. However, the curves superimposed well after 2 min post injection, where the POP-IF are based on the individual blood measures.

All combined data from 36 FLT dynamic PET scans were available and analysed. The population average derived IFs, as expected, did not produce the same initial peak height (from 0-2 min) as that of the original obtained with full arterial sampling (Figure 2). However, after approximately 2 min, the POP-IF curves were almost undistinguishable to that of the full arterial sampling method.

Due to these observations, the area under the plasmatic curve (AUC) was divided in two parts: AUC-1 was calculated between 0.0 and 2.5 min, and AUC-2 was calculated from the first individual discrete sample (taken at 2.5 min for all protocols) to the end of the scan. The mean absolute percentage difference (± standard deviation) between the full arterial sampling IF and the POP-IF was 30% (± 16%) for the peak height (highest point of the IF), 16% (± 20%) for the peak position (position with time on the × axis) and 13% (± 18%) for the area under the peak (AUC-1). However, the AUC-2 mean absolute percentage difference between the full arterial sampling IF and the POP-IF was 1.5% (± 0.6%) using POP-IF7, 3.5% (± 2.5%) using POP-IF5 and 5.3% (± 2.6%) using POP-IF3, indicating that the POP-IFs provide a reasonable approximation of the full arterial sampling IF in the latter part of the curve.

### Quantification of FLT uptake using the population based input functions

**Figure 3:**
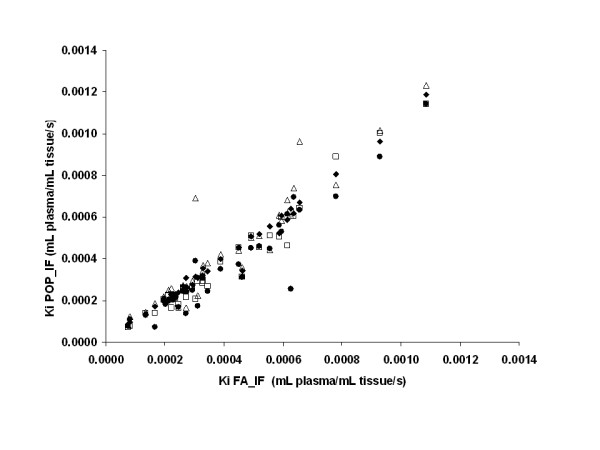
**The correlation between FA-IF and POP-IFs modified Patlak K_i_**. Correlation between modified Patlak K_i _using FA-IF and the Ki obtained using POP-IF7 (circles, r^2 ^= 0.98), POP-IF5 (squares, r^2 ^= 0.96), POP-IF3 (crosses, r^2 ^= 0.88) and POP-IF3M (triangles, r^2 ^= 0.85).

We investigated the accuracy of FLT uptake quantification obtained with the three limited blood sampling protocols described. Using the modified Patlak modelling method [5,28], the mean absolute percentage difference between Ki values obtained using FA-IF and Ki obtained using POP-IF7 was 5.0% (± 5.2%), was 10.3% (± 9.1%) using POP-IF5, 12.8% (± 13.1%) using POP-IF3, and 14.8% (± 14.3%) using POP-IF3M. High correlation (Figure 3) was found between Ki values obtained by the full arterial sampling, and Ki obtained using the population-based input with seven discrete samples (POP-IF7, R^2 ^= 0.98), five discrete samples (POP-IF5, R^2 ^= 0.96), three discrete samples (POP-IF3, R^2 ^= 0.88) and three discrete sample without metabolite correction (POP-IF3M, R^2 ^= 0.85). In patients with breast cancer, the standard deviation of K_i _estimated from FLT reproducibility studies (test-retest without treatment) using Patlak graphical analysis and FA-IF was 15% [24]; hence, the estimation error obtained using the new POP-IFs with limited sampling was within the intrinsic estimation error of Ki.

**Figure 4:**
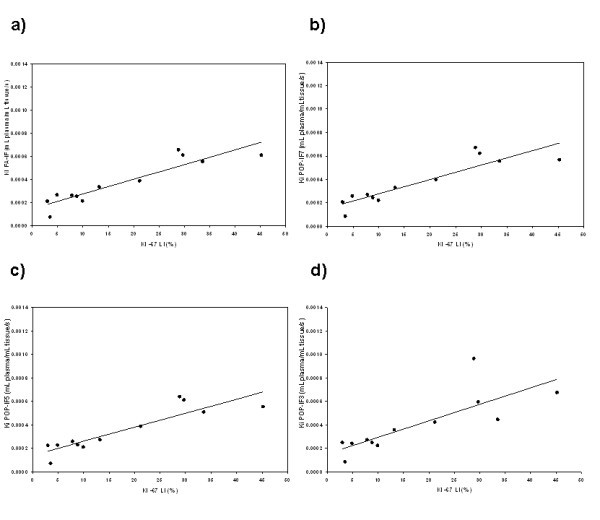
**The correlation between Ki-67 LI and modified Patlak Ki**. Correlation between Ki-67 LI and modified Patlak Ki using (**a**) FA-IF (R^2 ^= 85), (**b**) POP-IF7 (R^2 ^= 81), (**c**) POP-IF5 (R^2 ^= 80) and (**d**) POP-IF3 (R^2 ^= 64).

FLT is used as a marker of proliferation, and retention of FLT is believed to be related to the phosphorylation of the radiotracer by TK1. Good correlation was found between POP-IF Ki values and a standard marker of proliferation, Ki-67: using seven discrete samples (POP-IF7, R^2 ^= 0.81), using five discrete samples (POP-IF5, R^2 ^= 0.80), using three discrete samples (POP-IF3, R^2 ^= 0.64) and using three discrete samples without metabolite correction (POP-IF3M, R^2 ^= 0.60, not shown), versus R^2 ^= 0.85 for FA-IF as shown in Figure 4. These values are superior or comparable to the correlation found between Ki-67 and SUV values in the same data set (R^2 ^= 0.62) [5]. For the patients who completed two pre-treatment scans, regression analysis showed a good test-retest correlation in all POP-IF protocols, namely R^2 ^= 0.92 for POP-IF7, R^2 ^= 0.89 for POP-IF5, R^2 ^= 0.90 for POP-IF3, R^2 ^= 0.81 for POP-IF3M, versus R^2 ^= 0.94 for FA-IF.

FLT-PET net flux quantification may be needed to determine the effect of new drugs on tumour proliferation as semi quantitative measures like SUVs may be affected by changes in the blood flow. Kinetic quantification normally requires continuous concurrent monitoring of radioactivity in the blood. These measurements are invasive, costly and difficult to implement in routine clinical practice. In this investigation, we calculated the physiologically relevant rate constant K_i _in tumours by employing a limited discrete sampling approach without continuous arterial sampling. This has been possible by constructing a population input function (POP-IF) based on a leave-one-out cross-validated average of the pre-treatment scans subset for the initial part of the curve and on the individual plasma and metabolites data for the latter part. Although this population-based method could lead to inaccurate estimates of the rate constants describing initial kinetic events such as delivery of FLT, because of the larger individual variability of the initial peak of the plasma curves, we noted that the POP-IF curves (corrected either by seven, five or three individual discrete samples) were comparable to the full arterial IF curve approximately 2 min after injection of FLT, since in this part of the curve, they are based on the modelling of individual blood and plasma measurements. For this reason, the reduced sampling protocol did not affect the estimation of net transfer constant K_i _that depends on the K_1_/k_2 _ratio (with errors in K_1 _likely reflected in k_2 _and normalised in their ratio) and on the phosphorylation rate constant k_3 _that is reflected on the late points in the curve. Note that K_i _was estimated using the established modified Patlak's graphical method. This simplified procedure does not derive the two compartment model's rate constants individually. We expect that a complete solution of the non-linear model would be more dependent on IF accuracy. The POP-IFs generated results in good agreement with the original FA-IFs for both pre-treatment and post-treatment scans. Treatment *per se *did not affect the IF. There was a high correlation between the values of K_i _derived using FA-IF and all the POP-IF protocols evaluated in this study (R^2 ^= 0.85-0.98). The POP-IF Ki were also correlated with the percentage of Ki-67 positive cells (R^2 ^= 0.81-0.60) and performed fairly well in reproducibility test (R^2 ^= 0.81-0.92). The estimation errors increased with decreased number of samples because of the less accurate modelling of the individual blood and plasma curves.

This approach could be used for other ligands that are metabolised. To this end, the degree of metabolism will affect the robustness of the approach, with estimates of IF for minimally metabolised tracers being the most robust, particularly when POP-IF3M is used as an estimate.

FLT image-derived input functions reported similar results in reproducibility tests, correlation with Ki-67 and comparison with FA-IFs [21-23]; however, not all tumours will be located in regions with a prominent artery or heart, careful post processing of the raw data may be needed for partial volume and spill over correction given the size of the vessels, which additionally renders these input functions more sensitive to noise and movement. Blood sampling may, in any case, be necessary for metabolite correction of plasma data in compartmental based models.

Venous samples or arterialised venous samples will be easier for patients to tolerate, and compliance is likely to be higher. Considerable effort has been directed at investigating FLT pharmacokinetics in venous and arterial blood. It has been shown that the concentration of FLT in venous plasma samples is systematically marginally higher than the concentration in arterial plasma samples, but this was not found to make a statistically significant difference in analyses [20,21]. Shields et al. showed that [14] venous input functions exhibited good correlation with the aortic image-based IF, and venous samples have been used to correct aortic image-based IF for plasma over blood ratio and plasma metabolite fraction [22]. These studies justify the use of venous blood measurements of FLT-PET for the purpose of K_i _quantification. For these reasons, we expect that population input functions derived using plasma and metabolite correction from venous blood samples, a much more convenient method in clinical settings as opposed to arterial sampling, may yield similar accuracy of Ki values and could make FLT kinetic estimation in tumours even more widely applicable.

## Conclusions

Population-based input functions scaled using limited sampling protocols (consisting of seven, five or three discrete blood measurements), yielded results comparable to the ones obtained using continuous arterial sampling. Consequently K_i_, the physiologically relevant rate constant that quantifies tumour radiotracer retention in FLT-PET scans, may be estimated with a small loss of accuracy using a short (60 min) scan protocol without continuous arterial sampling.

The appropriateness of a population input function and highly reduced blood protocol approach will depend on the purpose of the FLT-PET study in question, as the practical advantages of reducing the number of sampling points is offset by an increasing loss of accuracy in the Ki estimation. The lack of metabolite correction reduced the accuracy of the Ki estimation further; however, the correlation with Ki-67 was still comparable with the one obtained with SUV values.

## Competing interests

The authors declare that they have no competing interests.

## Authors' contributions

LR and EOA designed the study. KBC, LMK and CRC carried out the clinical aspects of the study. LR, KBC and FET carried out the analytical aspects of the study including statistical analysis and modelling. LR, KBC, EOA drafted the manuscript. All authors read and approved the final manuscript.

## Appendix

Schematic derivation of the POP-IFs used in this study.

(1) $$\begin{array}{c} \text{POP}\text{\_}\text{IF7}\left(t\right)=\text{Cp}\left(t\right)\cdot \left(1-\frac{x_{1}+x_{2}\cdot t_{\text{parent}}}{{\left(x_{3}/t_{\text{parent}}\right)}^{x_{4}}+1}\right)\\ \text{where}\\ \text{Cp}\left(t\right)=\text{ID}\cdot \text{AVGB}\left(t\right)\cdot \left(x_{\text{1}}\cdot \text{exp}\left(-x_{2}\;t_{\text{pob}}\right)+x_{3}\right)\;\;\;0\leq t\leq \tau \\ \text{Cp}\left(t\right)=\;\text{spline}\left(t_{\text{plasma}}\right)\;t>\tau \;\;\;\;\; \end{array}$$

(2) $$\begin{array}{c} \text{POP\_IF5}\left(t\right)=\text{Cp}\left(t\right)\cdot \left(1-\frac{x_{1}+x_{2}\cdot t_{\text{parent}}}{{\left(x_{3}/t_{\text{parent}}\right)}^{x_{4}}+1}\right)\\ \text{where}\\ \text{Cp}\left(t\right)=\text{ID}\cdot \text{AVGB}\left(t\right)\cdot \left(x_{\text{1}}\cdot \text{exp}\left(-x_{2}\;t_{pob}\right)+x_{3}\right)\;\;\;0\leq t\leq \tau \\ \text{Cp}\left(t\right)=\;\text{spline}\left(t_{\text{plasma}}\right)+x_{4}\;\;t>\tau \end{array}$$

(3) $$\begin{array}{c} \text{POP\_IF3}\left(t\right)=\text{Cp}\left(t\right)\cdot \left(x_{\text{1}}\cdot \text{exp}\left(-x_{2}\;t_{\text{parent}}\right)+x_{3}\right)\\ \text{where}\\ \text{Cp}\left(t\right)=\text{ID}\cdot \text{AVGB}\left(t\right)\cdot \left(x_{\text{1}}\cdot exp\left(-x_{2}\;t_{\text{pob}}\right)+x_{3}\right)\;\;\;0\leq t\leq \tau \\ \text{Cp}\left(t\right)=\left(x_{\text{1}}\cdot \text{exp}\left(-x_{2}\;t_{\text{plasma}}\right)+x_{3}\cdot \text{exp}\left(-x_{4}\;t_{\text{plasma}}\right)+x_{5}\right)\;\;t>\tau \end{array}$$

(4) $$\begin{array}{c} \text{POP\_IF3M}\left(t\right)=\text{Cp}\left(t\right)\\ \text{where}\\ \text{Cp}\left(t\right)=\text{ID}\cdot \text{AVGB}\left(t\right)\cdot \left(x_{\text{1}}\cdot \text{exp}\left(-x_{2}\;t_{\text{pob}}\right)+x_{3}\right)\;\;\;0\leq t\leq \tau \\ \text{Cp}\left(t\right)=\left(x_{\text{1}}\cdot \text{exp}\left(-x_{2}\;t_{\text{plasma}}\right)+x_{3}\cdot exp\left(-x_{4}\;t_{\text{plasma}}\right)+x_{5}\right)\;\;t>\tau \end{array}$$

where AVGB(t) is the average dose-normalised blood function calculated over all dose normalised pre-treatment blood input functions excluding the blood data of the patient to be analysed, ID is the injected dose, *x_i _*are the generic parameters of function interpolation, *t*_pob _are the measurement of plasma over blood radioactivity concentration ratio, *t*_plasma _are the measurements of plasma radioactivity concentration, *t*_parent _are the measurements of the plasma fraction of unmetabolised FLT and *τ *is a value close to150s.
